# Targeted Therapy and Mechanisms of Drug Resistance in Breast Cancer

**DOI:** 10.3390/cancers15041320

**Published:** 2023-02-19

**Authors:** Briana Kinnel, Santosh Kumar Singh, Gabriela Oprea-Ilies, Rajesh Singh

**Affiliations:** 1 Department of Microbiology, Biochemistry, and Immunology, Morehouse School of Medicine, Atlanta, GA 30310, USA; 2 Department of Pathology & Laboratory Medicine, Winship Cancer Institute, Emory University School of Medicine, Atlanta, GA 30322, USA; 3 Cancer Health Equity Institute, Morehouse School of Medicine, Atlanta, GA 30310, USA

**Keywords:** drug resistance, MDR, HER2, iNOS, aryl hydrocarbon receptor, PTK6, CDK4/6, Wnt/β-catenin

## Abstract

**Simple Summary:**

Internationally, in women, breast cancer (BC) is the most common cancer type and is the leading cause of cancer-related death. There are two main histological classifications of BC, carcinoma in situ and carcinoma invasive. BC is further histologically subclassified as ductal carcinomas and lobular carcinomas. Once diagnosed, BC is typically treated based on the molecular subtype of cancer. The subtypes are based on the presence or absence of hormone receptors progesterone receptor (PR), estrogen receptor (ER), and human epidermal growth factor receptor 2 (HER2). The main molecular subtypes of BC include Luminal A (ER+, PR+, HER2-), Luminal B (ER+, PR+ HER2+), HER2 enriched (ER−, PR−, HER2+), and basal-like/ triple-negative BC (TNBC). People with hormone-positive (ER+ and or HER2+) BC are typically treated with endocrine therapy and chemotherapy, making them easier to treat. TNBC, on the other hand, is more challenging to treat due to the lack of hormone receptors and the aggressiveness of this form of cancer. Unfortunately, most therapies used to treat BC can often lead to drug resistance, relapse, and metastasis to other body parts. Due to its complexity and many mechanisms, it is difficult to overcome drug resistance. However, further research into potential drug targets and their relationship with drug resistance could help circumvent BC drug resistance.

**Abstract:**

Breast cancer is the most common cause of cancer-related death in women worldwide. Multidrug resistance (MDR) has been a large hurdle in reducing BC death rates. The drug resistance mechanisms include increased drug efflux, enhanced DNA repair, senescence escape, epigenetic alterations, tumor heterogeneity, tumor microenvironment (TME), and the epithelial-to-mesenchymal transition (EMT), which make it challenging to overcome. This review aims to explain the mechanisms of resistance in BC further, identify viable drug targets, and elucidate how those targets relate to the progression of BC and drug resistance.

## 1. Introduction

Internationally, cancer is one of the leading causes of death. By 2040, the global cancer burden is expected to rise 47% from 2020 rates [1]. As time progresses, the incidence of cancer has increased due to the aging and growth of populations and the changes in the prevalence of cancer risk factors. In women, BC is the most common type of cancer. According to the GLOBOCAN 2020, female breast was the most commonly diagnosed cancer, with an estimated 2.3 million new cases making up 11.7% of newly diagnosed cases [1].

BC is most commonly located in the milk ducts (ductal carcinoma) or the lobules (lobular carcinoma) [2]. BC can be referred to as in situ, meaning cancer has not grown into the rest of the breast, or invasive, which means the BC has infiltrated into the surrounding breast tissue [3]. Invasive ductal carcinomas account for 70–80% of all BC [2]. BC is further categorized into molecular subtypes based on the presence or absence of hormone receptors progesterone receptor (PR), estrogen receptor (ER), and human epidermal growth factor receptor 2 (HER2). The main subtypes are Luminal A (ER+, PR+, HER2−), Luminal B (ER+, PR+ HER2+), HER2 enriched (ER−, PR−, HER2+), and basal-like/ triple-negative BC (TNBC) [4]. Due to BC heterogeneity, BC treatment has moved from a one-size-fits-all approach to seeking more targeted therapy options. Currently, BC is treated based on tumor subtype [5]. Patients with ER+ tumors are typically treated with endocrine therapy and sometimes chemotherapy [6]. HER2+ tumors, on the other hand, are treated with monoclonal antibodies and tyrosine kinase inhibitors [7]. TNBC is typically more challenging to treat because it is not ER+, PR+, or HER2+. Because of this, chemotherapy is typically the therapy applied [8]. However, additional therapy options are being investigated, such as targeting the epidermal growth factor receptor (EGFR), androgen receptor (AR), poly (ADP-ribose) polymerase (PARP), and vascular endothelial growth factor receptor (VEGF) [9]. Other BC treatments being explored include cyclin-dependent kinase 4/6 (CDK 4/6) inhibitors, microRNAs (miRNAs), immunotherapy, clustered regularly interspaced short palindromic repeats (CRISPR), tyrosine kinase inhibitors (TKIs), PI3K inhibitors, drug repurposing, electrochemotherapy, surgery, and nanotechnology [9,10]. Table 1 displays the clinical trials for various types of BC treatments. Although technological advances have led to many breakthroughs in cancer treatments, resistance is still a major stumbling block in traditional and novel BC therapies. Because of this, drug resistance mechanisms must be investigated to detect further ways to combat drug resistance, leading to better health outcomes.

## 2. Drug Resistance

**Figure 1:**
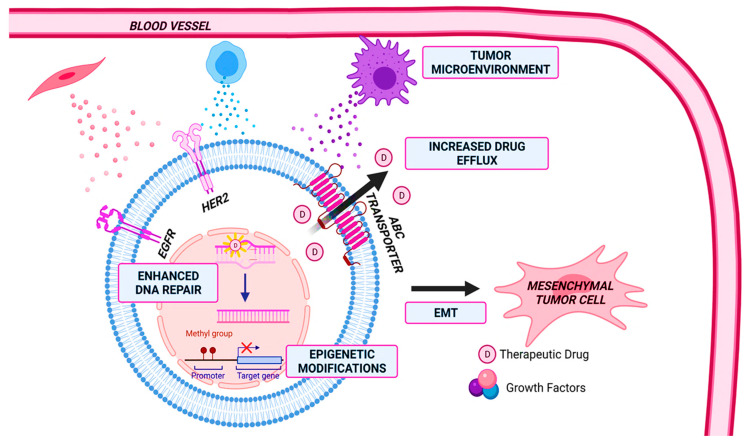
Mechanisms of drug resistance in breast cancer. These mechanisms include changes in the tumor microenvironment, enhanced DNA repair, epigenetic modifications, epithelial-to-mesenchymal transition (EMT), and increased drug efflux. Figure created with Biorender.com.

In cancer, drug resistance is responsible for up to 90% of deaths [3]. Multidrug resistance (MDR) hinders the efficacy of chemotherapeutic drugs, often leading to relapse and metastasis. Innate and acquired resistance each account for about 50% of patients with drug resistance [11]. Intrinsic resistance occurs before treatment and can be caused by genetic mutations, the proliferation of pre-existing insensitive subpopulations such as cancer stem cells, and the activation of inherent pathways that defend against toxic exogenous compounds [12]. On the other hand, acquired resistance can result from the activation of proto-oncogenes, alterations in gene expression due to mutations or epigenetic markers, and changes in the tumor microenvironment after treatment [12]. There are several mechanisms of resistance in BC. This includes increased drug efflux, enhanced DNA repair, senescence escape, epigenetic modifications, tumor heterogeneity, TME, and epithelial-to-mesenchymal transition (EMT) [12,13,14]. Mechanisms of drug resistance in BC are shown in Figure 1.

### 2.1. Increased Drug Efflux

The decrease in intracellular drug accumulation due to increased drug efflux is a significant factor in chemotherapy resistance [15]. The ATP-binding cassette (ABC) transporter subfamily plays a substantial role in drug efflux. Forty-eight ABC genes are grouped into seven subfamilies (ABCA, ABCB, ABCC, ABCD, ABCE, ABCF, and ABCG) [14,16]. ABCB1, ABCC1, and ABCG2 have been linked explicitly to MDR [14,17]. ABCB1, also known as MDR1 or P-glycoprotein (P-gp), can be expressed in normal tissues; however, it is overexpressed in many cancers [12]. In breast tumors, ABCB1 expression varies between individuals [18]. Overexpression of ABCB1 has been correlated with chemoresistance in several different cancers, including breast, kidney, colon, adrenal, pancreas, liver [19,20], prostate [21,22], and ovarian [23]. For example, paclitaxel-resistant SKBR3 and MCF-7 BC cells significantly overexpressed the ABCB1 protein and were partially desensitized following the silencing of ABCB1 [24]. Epigenetics often plays a significant role in the expression of ABCB1. CpG island hypermethylation of the distal promoter of ABCB1 is suggested to be linked to a reduction in ABCB1 transcript expression and increased overall survival in ovarian and BC patients [25]. ABCG2, the BC resistance protein, plays a vital role in BC therapy resistance and is a marker of cancer stem cells (CSCs) [26]. ABCG2 is capable of transporting both positive and negatively charged drugs. In TNBC, ABCC1 and ABCG2 are overexpressed compared to other BC subtypes [26].

### 2.2. Enhanced DNA Repair and Senescence Escape

DNA damage is the primary mechanism for many cancer therapeutics, such as cisplatin, 5-fluorouracil, gemcitabine, and methotrexate [27]. DNA damage response (DDR) leads to DNA repairs that can cause resistance by repairing the DNA lesions. Targeting major players in the DDR is a possible way to combat resistance. Poly (ADP-ribose) polymerase (PARP) plays a significant role in the DDR pathway. The use of PARP inhibitors to fight BRCA-deficient tumors has shown the potential of novel inhibitors of DNA repair proteins [28]. A combination therapy clinical study has looked into the use of PARP inhibitor Niraparib and anti-PD-1 antibody pembrolizumab in patients with TNBC or ovarian cancer (NCT02657889) [29]. However, PARP inhibitors can ultimately lead to innate and acquired drug resistance [30]. PARP and its relationship with DDR and resistance are further explained in further sections of this review. Another viable treatment option to combat DDR-related resistance is through the inhibition of DNA repair kinases. It has been demonstrated that inhibitors that target DNA damage repair kinases increased doxorubicin (DOX)-induced apoptosis in previously DOX-resistant BC cells [31]. DDR plays a complicated role in cancer treatment because DDR is essential for maintaining genetic stability. Because of this, continued research into more specific inhibitors is imperative to reduce the adverse effect on normal tissues and cells.

Senescence is also a known consequence of chemotherapeutic drugs. Senescence can be defined as permanent cell proliferation arrest. Three major stimuli that can trigger senescence include excessive mitogenic signaling by active oncogenes, telomere shortening, and non-telomeric DNA damage [32]. Over time, cancer cells with therapy-induced senescence can gain CSC-like properties, leading to the escape of senescence and tumor progression [33].

### 2.3. Epigenetic Modifications

Gene silencing via hypermethylation at CpG sites, overexpression via hypomethylation of oncogenes, histone modifications, and non-coding RNA expression alterations can influence chemotherapy drug tolerance [28]. Resistance can be acquired during targeted therapies via alterations of drug targets due to the mutation of the target proteins or alterations in expression levels due to epigenetic changes. Demethylation of the promoter region of an oncogene could potentially result in drug resistance because of increased gene expression. At the same time, hypermethylation of DNA repair genes is known to lead to cell cycle arrest inhibition. Tamoxifen (TAM), commonly used for ER+ BC patients, is an example of a targeted therapy that can lead to acquired resistance. TAM resistance varies by case; however, decreased ER expression can be due to mutation and epigenetic alterations, which are known causes [34]. In addition, microRNAs (miRNAs) are small non-coding RNAs that have been recognized as epigenetic modulators affecting gene expression via post-transcriptional modifications [28]. miRNA-27b-3p, miRNA-21, and miRNA-134 play a role in BC resistance by influencing genes related to resistance, cell cycle, cell proliferation, and apoptosis [34,35,36].

The Food and Drug Administration (FDA) has approved the use of epigenetic drugs such as DNA methylation inhibitors (DMNTi), 5-azacitidine, and 5-aza-2′-deoxycytidine (decitabine; DAC), as well as histone deacetylase inhibitors (HDACi), such as vorinostat, belinostat, romidepsin, and panobinostat [37,38,39]. In the Phase II National Cancer Institute/Stand up to Cancer Study, 5-azacitidine and entinostat were used as a combination epigenetic therapy in advanced BC [40]. The study showed that the combination was well tolerated and suggested that epigenetic therapy may be advantageous for some women. However, the use of DAC to treat BC has been shown to possibly increase the risk of metastasis by enhancing SIPA1 expression, which could facilitate the EMT of cancer cells [41]. Several clinical trials are also looking into combination treatments using vorinostat to treat BC (NCT00616967, NCT03742245, NCT04190056) [42]. Belinostat has been shown to work synergistically to decrease the proliferation and metastasis of MDA-MB-231 cells [43]. Belinostat is also used in clinical trials for metastatic BC, metastatic castration-resistant prostate cancer, and metastatic ovarian cancer (NCT04703920, NCT04315233). In combination with cisplatin and nivolumab, Romidepsin has also been clinically tested in patients with metastatic TNBC (NCT02393794) [44]. According to a recent study in BC, Panobinostat can possibly regulate tumor growth through exosomal secretions via the Vps34/Rab5C pathway [45].

### 2.4. Cancer Stem Cells and Tumor Heterogeneity

Within solid BC tumors, there is a wide variety of subpopulations of cancer cells with unique gene expressions that can affect the level of susceptibility to cancer therapies. Due to their self-renewal properties, CSC plays a vital role in tumor heterogeneity, relapse, and metastasis [26]. Evidence has shown that breast CSC are resistant to conventional therapies, causing them to accumulate after treatment [26]. One way CSC can be identified is through CSC biomarkers. CD133 has been identified as a marker in breast tumors, and high expression of CD133 has been linked to drug resistance [46]. Deregulated developmental signaling pathways, such as the Wnt/β-catenin, hedgehog (Hh), Notch, and JAK/STAT pathways, also play significant roles in the development and regulation of CSC as well as promote therapy resistance [46,47,48]

The mechanisms applied by CSC to evade death are still not entirely known. What is known is that CSC is renowned for remaining dormant for periods, and as previously stated, they express high levels of ABC transporters leading to increased drug efflux [26]. Therapy resistance has also been linked to the dormancy of CSC and to the avoidance of therapy-induced apoptosis via the Rho protein family. CSC can mediate resistance through dormancy by remaining in the G0 phase of the cell cycle, thus avoiding the effects of chemotherapy and irradiation, which are mainly effective against cells that are proliferating [49]. Tumor dormancy is believed to be a very important mechanism of therapeutic resistance [49]. Rho is a small GTPase that plays an important role in cell polarity, cell migration, cell cycle progression, and cancer progression [50,51]. Activation of the Rho- Rho-associated protein kinase (ROCK) pathways promotes survivin expression [46]. There is a positive correlation between RhoC and ALDH, a BC stem cell marker [52]. RhoC expression in breast tumor tissue was found to be higher after chemotherapy when compared to expression before treatment and in MCF-7 cells [53].

### 2.5. Tumor Microenvironment

The tumor microenvironment (TME) consists of surrounding blood vessels, immune cells, fibroblasts, signaling molecules, and the extracellular matrix. These components contribute to cancer’s hallmarks and intrinsic and acquired resistance [11].

Hypoxia is a significant characteristic of the TME that plays a vital role in cancer progression. Hypoxia is known to induce various mechanisms associated with the CSC phenotype, angiogenesis, drug efflux via increased expression of ABCC1 and ABCG2, modulation of senescence and apoptosis, metastasis, and genomic instability [11,26]. Hypoxia leads to the production of hypoxia-induced factor (HIFs), a transcription factor that responds to low oxygen levels mediating the effects of hypoxia. HIF-1 promotes epithelial–mesenchymal transition (EMT) and is a hallmark of TNBC [26]. HIF-1α is also said to contribute to drug resistance in ER+ BC cell lines [54].

Another way hypoxia can induce resistance is by generating a “reversed pH gradient” [11]. Cancer cells have a more acidic extracellular environment (pH 6.5–7.1) than normal tissues. The decrease in extracellular pH leads to the ion trapping of weak-base anticancer drugs, which leads to poor distribution [11]. Proton pump inhibitors are one example of a therapeutic strategy that can increase extracellular pH, making it a suitable target for resistance treatment. The production of growth factors in and around the cell greatly influences cancer progression. In BC, increased activation of extracellular matrix (ECM) proteins and protein kinase C (PKC) via the receptor binding of growth factors is associated with resistance. The remodeling of the ECM by factors such as matrix metalloproteins (MMPs)-2, -9, -11, and -14 leads to cancer progression and metastasis due to ECM degradation [55]. The production of growth factors such as vascular endothelial growth factor A (VEGF-A) by tumor-associated macrophages encourages tumor invasion by using various mechanisms, such as inducing angiogenesis [56]. These factors may originate from nearby adipocytes and fibroblasts [56,57].

### 2.6. Epithelial to Mesenchymal Transition (EMT)

In normal cells, EMT is a process that is involved in embryonic development and wound healing; however, this process can be hijacked via various mechanisms, leading to tumorigeneses [54]. EMT plays an important role in therapeutic resistance, tumor invasion, and metastasis in cancer. During EMT, E-cadherin function is lost, leading to reduced cell–cell adhesion and apoptosis resistance [54]. The mechanisms by which EMT induces drug resistance is not fully understood; however, there has been some hypotheses presented. One theory is that because EMT and CSC share similar phenotypes, EMT may also have signaling pathways that enable therapeutic resistance, such as Wnt [11]. Another example of this is TGF-β. TGF-β, a cytokine subfamily of growth factors, is said to be involved in breast CSC regulation, EMT, and drug resistance [11,26]. It is also believed that there is a link between EMT transcription factors, such as Twist, Snail, Slug, ZEB, and FOXC2, and drug resistance. Increased RhoC expression through chemotherapeutic etoposide has also induced an EMT-like phenotype in BC cells, further emphasizing the association of CSC, EMT, and resistance [58].

## 3. Potential Targets and Resistance

### 3.1. Aryl Hydrocarbon Receptor (AhR)

The Aryl hydrocarbon receptor (AhR) is a ligand-activated transcription factor that belongs to the basic helix-loop-helix/Per-Arnt-Sim (Bhlh/PAS) family [59,60]. AhR is the only member of this family known to be activated by ligands [61]. These ligands can be endogenous and exogenous, exhibiting tissue-dependent antagonist or agonist activities [59]. The AhR was first primarily studied as an environmental sensor that mediates the effects of xenobiotic exposures [59,60,61]. A growing literature suggests that the AhR may also play a vital role in regulating various biological activities, such as mammary gland development. It can inversely play a role in pathological conditions such as BC [62].

When unbound to a ligand, the AhR forms a cytosolic complex with two molecules of heat shock protein 90 (Hsp90), prostaglandin E synthase 3 (p23), and a molecule of hepatitis B virus X-associated protein 2 (XAP-2) [63]. XAP-2, a 43-kDa immunophilin-like protein, also known as the AhR-interacting protein (AIP), plays a vital role in preventing AhR ubiquitination [64]. Hsp90 helps maintain a complex formation that results in high ligand-binding affinity while avoiding the binding of DNA recognition elements [65]. P23 also acts as a complex stabilizer that ensures the complex’s cytoplasmic localization [59].

**Figure 2:**
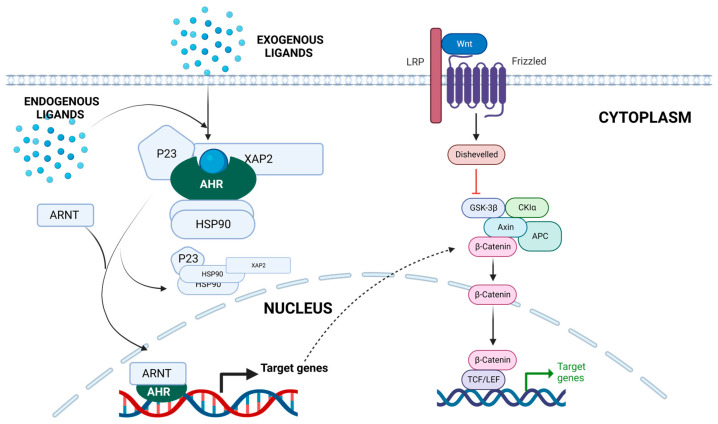
The Aryl Hydrocarbon Receptor (AhR) pathway. When inactive, the AhR is located in the cytoplasm and forms a complex with XAP2, also known as AIP, two HSP90 molecules, and p23. After ligand interaction, AhR translocates to the nucleus, interacting with ARNT to create a heterodimer. The heterodimer binds to the XRE and coregulators, leading to the transcription of target genes. AhR crosstalk with the Wnt pathway is also displayed. Figure created with Biorender.com.

As a result of ligand interaction, AhR is translocated into the nucleus, and its conformation is altered, revealing AhR nuclear localization signals [66]. The complex dissociates, and AhR forms a heterodimer with the AhR nuclear translocator protein (ARNT) [66]. The heterodimer formation allows the AhR to bind to specific DNA xenobiotic response elements (XREs), also known as the AhR-binding DNA consensus motif, 5′-TNGCGTG-3’ [67]. Genes with XRE in their promoter sequence, such as UDP-glucuronosyltransferase (UGT1A1), NAD(P)H quinone oxidoreductase 1 (NQO1), AhR repressor protein (AHRR), and cytochrome P450 1A1,1A2, and, 1B1 (CYP1A1, CYP1A2, and CYP1B1), can be transcriptionally activated by AhR DNA binding [68]. The induction of CYP1A1 results in the formation of ligand-based electrophilic intermediates that are vital for DNA adduct formation [69]. The AhR pathway is shown in Figure 2.

#### 3.1.1. AhR and BC

AhR mRNA is overexpressed in BC compared to healthy tissues, making it a viable drug treatment target [70]. Using ChiP-seq analysis, a study involving the genome-wide mapping of AhR binding sites in human BC cells identified up to 4000 AhR-bound regions [60]. In BC, the effects of AhR activation are context dependent. When exogenous ligands activate AhR, studies using different cell lines often result in varying results [61]. AhR can act as a tumor suppressor via tumor suppressor miRNA regulation [60]. It has also been previously reported that AhR blocks proliferation via AhR crosstalk with ER [71]. Small molecules that serve as AhR agonists have also been found to inhibit tumor invasion and metastasis in basal-like BC subtypes, which are known to resist endocrine therapy [71].

In contrast, clinical studies have shown that in environmental carcinogens, such as 2,3,7,8-tetrachlorodibenzo-p-dioxin (TCDD) and polycyclic aromatic hydrocarbons (PAHs), toxicity is mediated by AhR binding, and exposure can lead to diseases such as BC [72]. The tumor suppressor is often silenced in BC [73]. Loss of AhRR has been correlated to decreases in patient survival [73]. An in vivo investigation with AhRR transgenic mice showed that overexpression of AhRR can inhibit AhR-driven and inflammation-induced growth of BC [73]. The results also demonstrated that AhR knockout in human BC cells could increase chemotherapeutic-induced apoptosis and reduce mice tumor growth [73]. These findings suggest that under certain circumstances, AhR antagonists may also serve as a viable BC treatment option.

#### 3.1.2. AhR and Drug Resistance

Recent studies into AhR have shown that in BC, AhR can promote chemotherapeutic resistance in cancer stem cells and aggressive BC phenotypes via the Wnt5a/β-catenin signaling pathway [61]. In various BC cell lines, AhR plays a role in conferring apoptotic resistance. AhR is a key mediator of metabolizing enzymes and transporters [74]. Activation can lead to an increase in metabolizing enzyme gene expression [74]. This can contribute to the acceleration in the efflux of pharmaceutical compounds and decrease drug activity. One theory for this phenomenon is that because the AhR has evolved as a pro-survival factor when exposed to environmental stress, the AhR also can act as a defense against stress-induced apoptosis [75]. AhR can also influence drug-metabolizing enzymes (DMEs) and transporters by promoting the expression of MDR genes and MDR-associated proteins via other transcription factors [74]. AhR is, directly and indirectly, involved in cell cycle regulation, early gene induction, mitogen-activated protein kinase cascades, Rb protein function, and responses to matrix composition changes [74]. This suggests that AhR is a viable target for combating drug resistance.

AhR’s role in drug resistance, like its role in cancer, is variable. The chemotherapeutic drug TAM is widely used to prevent and treat steroid hormone receptor-positive BC; however, its use often leads to drug resistance [71,74]. AMD3100, a small molecule AhR antagonist, led to the inhibition of Tamoxife- resistant (TamR) MCF7 progenitor cells and inhibited the growth of TamR MCF7 xenografts in vivo. This occurs via AhR-mediated downregulation of CXCR4 signaling. Conversely, nontoxic AhR agonists, such as Aminoflavone (AF), a dietary flavonoid, induce tumor suppressor-like qualities [59,61,71,75]. To avoid negative outcomes due to toxicity and drug resistance, dietary flavonoids that target the AhR are being investigated for BC treatment options [1,3]. AF inhibits the expression of α6-integrin, suppressing the α6-integrin-Src-Akt signaling pathway and leading to reduced cell viability in TamR cells [71]. Cells with higher levels of α6-integrin expression were less sensitive to the effects of AF [71]. AF exhibited selectivity for ER-positive cell lines and was ineffective against MDA-MB-231 and Hs578T [59].

### 3.2. Inducible Nitric Oxide Synthase (iNOS)

**Figure 3:**
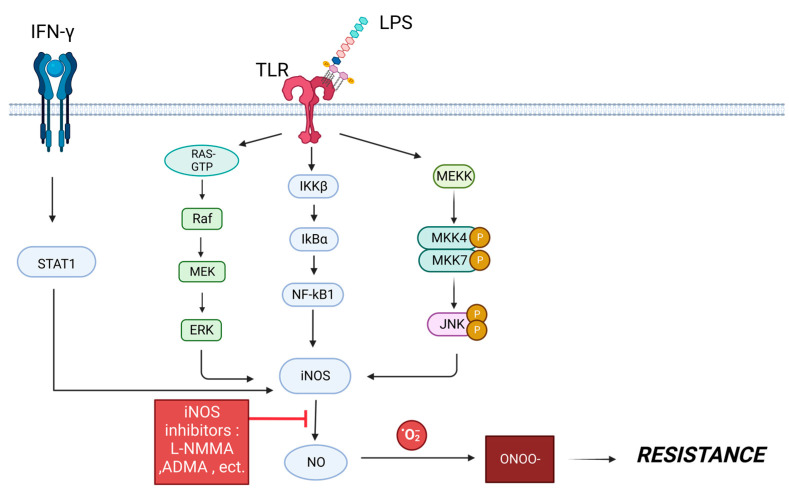
Signaling pathways that induce inducible nitric oxide synthase (iNOS) production, leading to nitrous oxide (NO) production. These pathways include the signal transducer and activator of transcription 1 (STAT1), Ras/Raf/MAPK, an inhibitor of nuclear factor kappa-B kinase (IKKβ), and c-Jun N-terminal kinase (JNK) pathways. NO reacts with oxygen free radicals to produce peroxynitrite, which can lead to drug resistance. NO production could potentially be reduced using iNOS inhibitors such as NG-monomethyl-L-arginine acetate (L-NMMA) and asymmetric dimethylarginine (ADMA). Created with BioRender.com.

By converting L-arginine to L-citrulline, the enzyme nitric oxide synthase (NOS) forms the free radical nitric oxide (NO) [76]. NO regulates various biological functions such as vascular relaxation, neurotransmission, and immune response facilitation. NOS has three isoforms, neuronal NOS (nNOS), inducible NOS (iNOS), and endothelial NOS (eNOS) [77]. Unlike nNOS and eNOS, iNOS activity is calcium independent, giving it the ability to produce higher amounts of NO [77]. In cancer, NO production can play a dual role, being both oncogenic and exhibiting some antitumor effects. The role of NO is concentration dependent [78]. Low NO doses of 10–300 nM, induced by spermine NONOate in MCF-7 BC cells, led to ERK phosphorylation and hypoxia-induced factor one alpha (HIF-1α), which can result in tumor progression [79]. At high concentrations above 300 nM, NO can lead to p53 phosphorylation, which is linked with the induction of apoptosis [79]. The effects of NO attention have also been found to depend on the expression patterns of the NOS isoforms, duration, timing, tumor microenvironment, and cell type [76]. The signaling pathways that induce iNOS are shown in Figure 3.

#### 3.2.1. iNOS and BC

In BC, iNOS expression is typically high [80,81,82]. Studies have demonstrated a correlation between high iNOS expression and poor health outcomes in BC [81,83]. In patients that are ERα-negative, high iNOS expression has been seen to cause poor survival via the induction of interleukin-8 (IL-8), CD44, c-Myc, and the activation of ETS-1 [81,84]. An Oncomine Cancer Microarray database analysis of iNOS expression showed that invasive TNBC has higher iNOS expression than non-TNBC [83]. Patients with invasive ductal breast carcinomas exhibited a correlation between increased iNOS expression and decreased survival at five years [83]. iNOS inhibition has been demonstrated to reduce the tumorigenicity of TNBC by impacting cell proliferation, migration, and CSC renewal [83]. In MDA-MB-231 L/G cells in mouse models, small molecule NOS inhibitor NG-monomethyl-L-arginine acetate (L-NMMA) led to decreased proliferation rates when used alone and in combination with docetaxel, indicating that it is a potential novel targeted therapy [83].

As previously mentioned, NO, a product of iNOS, plays a pivotal role in BC. NO, via various signaling pathways, is known to cause the proliferation and migration of tumors and angiogenesis [85,86]. NO concentrations, 100 and 300 μM DETA-NO were reported to induce the migration of MDA-MB-231 cells [87]. A recent study attempted to see the effects that hyaluronic acid (HA), an essential neutralizer of free radicals in tumor tissues, has on NO-induced migration of BC cell lines [88]. It was found that, when treated with DETA-NO and HA, a significant decrease in migration was observed in MDA-MB-231 cells after 12 h of culturing; however, the rise in NO levels negated the effects at 24 h [88]. When treated with different concentrations of HA in iNOS-transfected HCC1806 cells, no change in migration was observed, despite HA’s affinity to decrease the levels of intracellular NO [88]. Further studies into optimizing HA-based drug delivery vehicles are being conducted.

#### 3.2.2. iNOS and Resistance

High levels of iNOS are linked with cisplatin resistance in some cancer cells [89]. Cisplatin sensitivity is restored after iNOS attenuation [89]. In TNBC patients with attenuated iNOS expression, there was an average tumor volume reduction of 90.31% after neoadjuvant chemotherapy. Patients with increased iNOS expression or unchanged iNOS expression showed an average tumor volume reduction of 59.35% in response to neoadjuvant chemotherapy [89]. These findings suggest that iNOS attenuation can increase platinum-based neoadjuvant chemotherapy in TNBC patients [89].

NO reacts with oxygen radicals to form peroxynitrite, a strong oxidative and nitrosative agent [76]. In cancer cells, nitrosative signaling contributes to proliferation, metastasis, and resistance to therapy [76]. In animal models of photodynamic therapy, iNOS-derived NO increases led to resistance to cell death in breast and prostate tumors [76]. In a separate study of TNBC cells, MDA-MB-231 cells were treated with 10 or 30 µM of DETA NONOate, a NO donor; it was observed that the growth inhibitory effects of cisplatin were diminished [89]. These findings suggest that an exogenous increase in NO can potentially result in resistance in BC.

### 3.3. PI3K/Akt/mTOR Pathway

**Figure 4:**
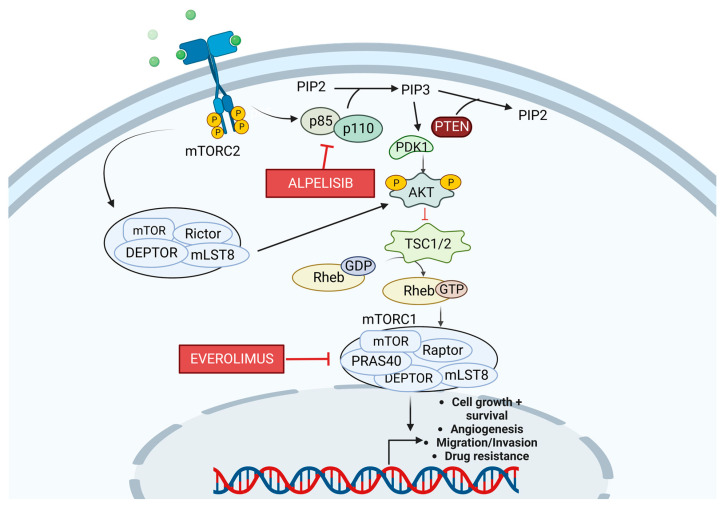
Diagram of the p85/p110 (PI3K) signaling pathway. PI3K leads to the formation of the second messenger, PIP3. PDK1 and AKT are then recruited to the membrane. PDK1 and mTORC2 phosphorylate AKT, fully activating it. AKT then phosphorylates TSC, which is a negative regulator of mTORC1. Alpelisib is a PI3K inhibitor, and Everolimus is an mTOR inhibitor. Created with BioRender.com.

Phosphoinositide 3 kinase (PI3K) is a heterodimer consisting of the regulatory subunit p85 and the catalytic subunit p110 [90]. P85 regulates the activation of p110 based on the presence of growth factors and receptor tyrosine kinases (RTKs). Activating the PI3K/Akt/mammalian target of the rapamycin (mTOR) pathway leads to cellular proliferation and plays a significant role in tumorigenesis [91]. Once activated, PI3K leads to the phosphorylation of phosphatidylinositol 4,5 bisphosphate (PIP2) to create phosphatidylinositol 3,4,4-triphosphate (PIP3). The tumor suppressor, PTEN, dephosphorylates PIP3 back to PIP2, which counteracts the PI3K signaling [90]. Formation of PIP3 leads to the phosphorylation of the kinase Akt. The activation of Akt leads to the inhibition of tuberous sclerosis 1/2 (TSC1/2), a tumor suppressor GTPase that activates the protein Rheb-GTP [92]. Because TSC is an inhibitor of mTOR, this results in the activation of the mTOR complex mTORC1 [93]. The mTORC1 complex consists of Raptor, mLST8, and the proline-rich Akt substrate 40 (PRAS40). mTORC1 activation leads to cell metabolism via GLUT-1 [94] and is also linked to protein synthesis, proliferation, and anti-apoptosis via action on the 40S ribosomal protein S6 kinase 1 (S6K1) and eukaryotic initiation factor 4E binding protein (4EBP1) [95]. The p85/p110 (PI3K) signaling pathway is shown in Figure 4.

#### 3.3.1. PI3K Pathway and BC

PI3KCA (phosphatidylinositol-4,5-bisphosphate 3-kinase, catalytic subunit alpha) mutations involving exons 9 and 20 are some of the most common mutations in BC [96]. According to the American Cancer Society, 30–40% of BCs have PIK3CA mutations. ER-positive tumors, especially the luminal A subtype, have been found to have the highest frequency of PI3KCA mutations. There are not many FDA-approved PI3K inhibitors. Piqray, also known as alpelisib, was the first FDA-approved PI3K inhibitor. Alpelisib is used alongside faslodex (fulvestrant), a hormone therapy already approved by the FDA, to treat HER2+/−, post-menopausal advanced BC patients with PI3KCA mutations [96]. A phase Ib/II trial was conducted to assess the efficacy of ribociclib + letrozole, alpelisib + letrozole, and ribociclib + alpelisib + letrozole treatment in ER+ HER2− BC patients. The trial’s findings showed that the ribociclib+ alpelisib+ letrozole combination caused a more consistent reduction of Ki-67, suggesting that targeting both CDK4/6 and PI3K may lead to better health outcomes for BC patients [97].

#### 3.3.2. PI3K and Resistance

The PI3K/Akt/mTOR pathway plays a vital role in BC endocrine, HER-2, and cytotoxic therapy resistance [96]. There is evidence that the estrogen receptor (ER) pathway can be activated by Akt when estrogen is not available [98]. Studies have demonstrated that inhibiting mTOR possibly plays a vital role in HR-positive and endocrine-resistant BC. Combination therapy using mTOR inhibitors alongside endocrine therapy has been shown to overcome endocrine therapy resistance. In HER2-overexpressing BC, the PI3K/Akt/mTOR pathway has also been implicated in trastuzumab resistance. Studies have demonstrated that PI3K pathway inhibitors work well with trastuzumab [99]. A phase 1b study involving Everolimus, a kinase inhibitor, in combination with paclitaxel and trastuzumab in trastuzumab-resistant patients had an overall response rate (ORR) of 44% and median PFS of 34 weeks [100]. The findings of these studies emphasize the benefit of combination therapies to overcome resistance.

### 3.4. Poly (ADP-Ribose) Polymerases (PARP)

**Figure 5:**
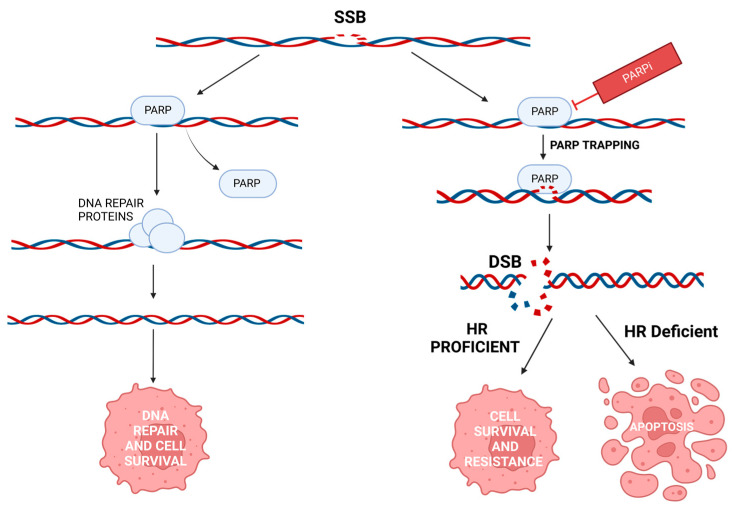
The effects of PARP binding after single-stranded DNA breaks. The binding of PARP1 helps detect the single-stranded break (SSB) and promotes the base excision repair (BER) mechanism for DNA repair. PARP must be released from the DNA for the repair to occur. When treated with PARP inhibitors (PARPi), PARP trapping occurs, leading to double-stranded breaks (DSB). When homologous recombination (HR) occurs, the DNA is repaired, and cell survival occurs. Created with BioRender.com.

Ten percent of BC patients have germline DNA mutations, while 90% of cases are caused by somatic, genetic, and epigenetic modifications [101]. There are at least 450 proteins believed to be involved in the DNA damage response (DDR) pathway [102]. PARP1, PARP2, and PARP3 are enzymes that are integral to the base excision repair (BER) pathway. PARP1 first binds to the damaged DNA. This allows NAD+ to bind to its active site, leading to the transfer of ADP-ribose moieties from NAD+ to target proteins via PARylation. PARylation mediates the recruitment of DNA repair effectors. PARP1 undergoes auto PARylation, leading to PARPs release from the DNA. Deregulating the DDR pathways can ultimately help cancer cells overcome the cytotoxicity due to chemotherapeutics and radiotherapy treatments [103]. The effects of PARP binding after single-stranded DNA breaks are shown in Figure 5.

#### 3.4.1. PARP and BC

BRCA1/2, known to play a vital role in homologous recombination repair (HRR), is mutated in about 30% of BC patients predisposed to BC due to family history [20]. Germline BRCA mutations are more likely to occur in specific populations than others. The loss of HRR is an essential condition for synthetic lethality. Synthetic lethality is the mechanism by which PARPi leads to cell death. PARP inhibitors (PARPi) prevent the release of PARPs from the DNA damage site, which removes the PARPs from their normal catalytic cycle [101,103]. Double-stranded breaks often form when single-stranded breaks are not repaired. HRR is the most accurate way to repair double-stranded DNA breaks. When HRR cannot be performed, non-homologous end joining (NHEJ) transpires. NHEJ can often accumulate genetic aberrations leading to genetic instability, cell cycle arrest, and apoptosis [101,103].

Olaparib and talazoparib are examples of PARPi that the FDA and EMA (European Medicines Agency) have approved to treat patients with locally advanced and metastatic BC in patients with deleterious or suspected deleterious germline BRCA (gBRCA)-mutated HER2-BC [101]. Many other PARPi are currently in clinical trials, such as niraparib, veliparib, and rucaparib [101]. There are also studies looking into using PARPi for early BC stages and in patients with non-gBRCA HRR gene mutations.

#### 3.4.2. PARP and Resistance

Although BRCA-deficient tumors are more sensitive to PARPi due to synthetic lethality, nearly 40% of BRCA1/2-deficient patients do not respond to PARPi [103]. Many patients that initially respond well to PARPi gain resistance over time to orally administered PARPi [103]. Homologous recombinant repair restoration and restoration of replication fork stability have been said to be the two leading causes of PARPi resistance [103]. PARPi functions by trapping PARP1 in its chromatin-bound state. An in vitro study showed that PARP1 is removed by ubiquitin-regulated p97 ATPase, suggesting that p97 plays a role in processing trapped PARP1 and PARPi resistance [104].

Fortunately, there are potentially several pathways to restore PARPi sensitivity. One option is to target ataxia-telangiectasia mutated and Rad3-related (ATR), one of three kinases that control most DNA damage responses [105]. The other two kinases are ataxia-telangiectasia mutated (ATM) and DNA-PK [106]. A study showed that Olaparib, a PARPi, combined synergistically with the ATR-inhibitor AZD6738 (ceralasertib) in vitro. This combination therapy resulted in selective cell death in ATM-deficient cells [107]. ATR inhibitors and PARPi have also been shown to be effective in treating cancers with BRCA1/2 mutations [106]. There is also a clinical trial underway investigating the use of Ceralasertib (AZD6738) and ATR inhibitors, alone and in combination with PARPi’s Olaparib or Durvalumab in patients with solid tumors (NCT03682289). Another potential avenue to overcome PARPi resistance is using cell cycle kinase inhibitors. WEE1 kinase prolongs the G2 phase of the cell cycle via the regulation of CDK1/2 phosphorylation [108]. In another in vitro study, for the WEE1 inhibitor AZD1775, the evidence showed that when combined with a PARPi, AZD1775 successfully modulates olaparib sensitivity in TNBC [109]. There is currently a Phase II clinical trial studying the safety and efficacy of therapies targeting DNA damage repair in conjunction with Olaparib compared to the use of Olaparib alone (NCT03330847). It has also been suggested that direct targeting of oncogene transcription could further sensitize cells to PARPi. Bromodomain containing 4 (BRD4) is linked to the transcription of several different oncogenes [110]. A study demonstrated that using BRD4 inhibitors in combination with PARPi resensitized cells to the PARPi treatment through the induction of homologous recombination deficiency [110].

### 3.5. Protein Tyrosine Kinase 6 (PTK6)

**Figure 6:**
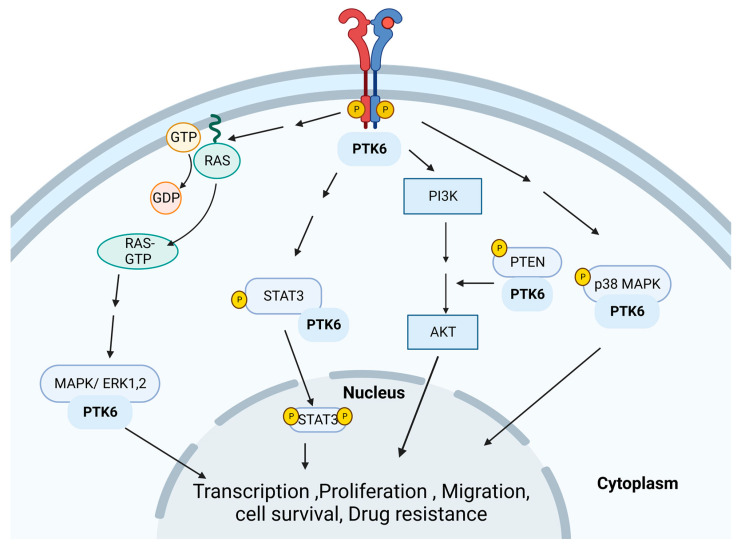
A diagram of PTK6 signaling. PTK6 activates several signaling pathways, such as JAK/STAT, PI3K, and MAPK, which enhance cell proliferation, invasion/migration, and resistance. Created with BioRender.com.

Protein tyrosine kinase 6 (PTK6), also known as the breast tumor kinase (Brk), is an intracellular non-receptor tyrosine kinase [111]. The PTK6 structure consists of SH2, SH3, a linker region, and catalytic domains [112]. PTK6 expression promotes oncogenic phenotypes such as enhanced proliferation, enhanced anoikis resistance, autophagy regulation, EMT, and invasion/ migration via the phosphorylation of HER2, PTEN, MAPK (ERK), p38 MAPK, STAT3 [113,114]. The pathway of PTK6 signaling is shown in Figure 6.

#### 3.5.1. PTK6 and BC

PTK6 is overexpressed in over two-thirds of BCs [115,116]. High PTK6 expression is linked to adverse health outcomes. ER+ and HER2+ cancers express the highest levels of PTK6 [117]. PTK6 expression levels have also been implicated in regulating sensitivity to targeted therapies. The overexpression of PTK6 in HER2+ MCF-10A cells was reported to suppress the effects of lapatinib treatment [113]. TNBC Xenograft models also demonstrated that PTK6 expression could increase sensitivity to chemotherapy agents’ DOX and paclitaxel [27]. Although PTK6 is also known as Brk, recent findings suggest that PTK6 kinase activity is not an oncogenic driver of BC [111,116,118]. Based on these findings, targeting the PTK6 kinase activity in BC may be a less effective therapeutic approach. When PTK6 levels were altered in a TNBC mouse model, little effect on the tumor volume was seen, but lung metastasis was reduced [111]. In TNBC cell models, it was found that PTK6 mediated cancer phenotypes via the AhR and ras homolog gene family, member A (RhoA) activation [111].

#### 3.5.2. PTK6 and Resistance

In a separate investigation of the regulators of anchorage-independent survival, PTK6 was identified as a key modulator of anoikis resistance in BC cells [117]. An in vitro study showed that the inhibition of PTK6 can lead to apoptosis of Lapatinib-resistant HER2+ BC cells through the enhancement of the pro-apoptotic factor Bim [113]. This study was the first to show that PTK6 inhibition is potentially a viable strategy to induce cell death in TKI-resistant HER2+ BC cells.

### 3.6. Cyclin-Dependent Kinase 4/6 (CDK4/6)

**Figure 7:**
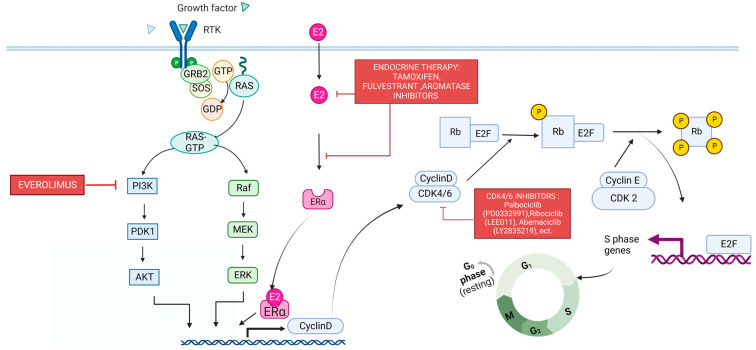
A diagram of how the binding of CDK4/6 relates to cell cycle progression. RAS signaling and estrogen receptor (ER) activation lead to the production of cyclin D. The binding of Cyclin D to CDK4/6 helps push the cell cycle from the G1 to S phase via Rb phosphorylation. The nodes at which current therapies target the pathway are included as well. Created with BioRender.com.

The loss of cell cycle regulation is a known hallmark of cancer. The cell cycle is typically regulated by the production and degradation of cyclins, cyclin-dependent kinases (CDK), and CDK inhibitors (CDKIs) [119]. CDK4/6, along with D-type cyclins (cyclins D1, D2, D3), regulates entry into the G1 phase during the cell cycle [120]. D-type cyclins are produced via RAS signaling, allowing for the binding and activation of CDKs 4 and 6 (CDK 4/6). The binding of cyclin D to CDK 4/6 initiates the phosphorylation of downstream substrates such as pRB and CDKIs p107 and p130. Phosphorylation of these substrates ultimately leads to the progression of the cell cycle [120]. The connection between CDK4/6 binding and cell cycle advancement is shown in Figure 7.

#### 3.6.1. CDK 4/6 and BC

In about 60% of BC, cyclin D1 is amplified [119]. High cyclin D1 and HER-2 expression are associated with recurrence and reduced response to TAM [119]. According to The Cancer Genome Atlas (TCGA), cyclin D1 amplification is preferential to luminal-type tumors, especially luminal B [121]. The use of oral CDK4/6 inhibitors such as palbociclib (PD 0332991), ribociclib (LEE011), and abemaciclib (LY2835219) are promising BC therapeutics [122].

In vitro studies have shown that using palbociclib can inhibit the cell cycle in ER+ and HER-2 amplified cells [119]. Studies have also demonstrated that Palbociclib efficacy is improved when combined with other therapeutics [122]. Palbociclib works synergistically with TAM and can enhance TAM sensitivity in ER+ and HER2+ cell lines [123]. In a phase III clinical trial, patients with untreated ER+, HER2- advanced BC that were treated with both Palbociclib and letrozole had a median progression-free survival (PFS) of 24.8 months [124]. Those treated with a letrozole+ placebo had a significantly lower median PFS of 14.5 months.

Ribociclib has also been shown to significantly inhibit tumor growth in a preclinical setting when used alone and in combination with other therapeutics, such as letrozole and fulvestrant, to treat ER+ xenograft models [125]. Data from a phase II clinical trial using ribociclib and letrozole to treat hormone +, HER2− BC patients showed a reduction in Ki-67 expression, phosphorylated Rb concentrations, and reduced the gene expression of CDK4, CDK6, cyclin D2, cyclin D3, and cyclin E1 [126]. A phase III trial using a combination of ribociclib and endocrine therapies, such as TAM, demonstrated significantly longer overall survival than endocrine therapies alone [127].

Abemaciclib is a small molecule inhibitor that inhibits CDK4 more effectively than CDK6 [128]. In a xenograft study, treatment with abemaciclib caused Rb phosphorylation inhibition, leading to cell cycle arrest [128]. In phase III trials, abemaciclib increased the efficacy of fulvestrant and NSAIDs such as anastrozole and letrozole [129,130].

#### 3.6.2. CDK 4/6 and Resistance

Although CDK 4/6 is a viable target for BC treatments, resistance to CDK 4/6 inhibitors is a significant barrier to their use. Non-luminal/basal subtypes were the most resistant to Palbociclib [119,123]. In ER+ MCF-7 cells, treatment with abemaciclib led to the production of clones, with CDK6 amplification leading to abemaciclib resistance [131]. Another potential resistance mechanism in metastatic BC patients is the loss of Rb1 expression after exposure to CDK4/6 inhibitors [132]. Because the downregulation of Rb is common in TNBC, CDK4/6 inhibitors alone may not be an ideal treatment for TNBC patients [133]. Other suggested resistance mechanisms include the amplification of cyclins and upstream signaling pathways [134]. CDK4/6 inhibitors, in combination with endocrine therapies, have successfully overcome resistance in HR+ patients [134].

### 3.7. Wnt/β-Catenin Pathway

**Figure 8:**
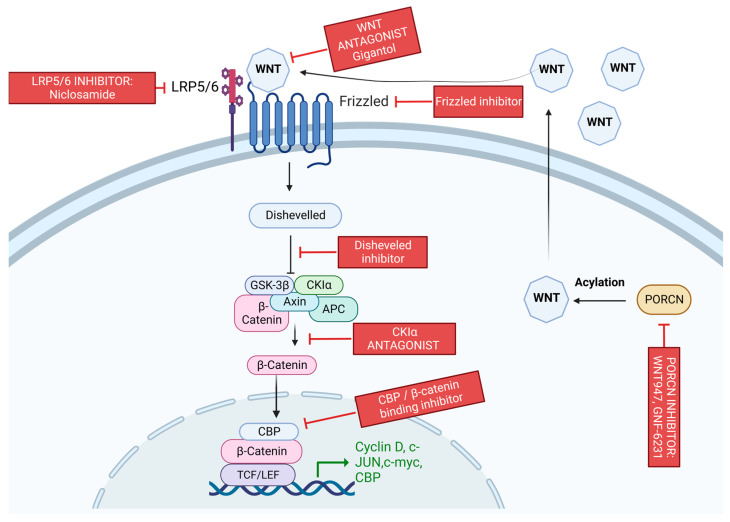
A diagram of the Wnt signaling pathway. The binding of Wnt to its receptors Frizzled and LRP5/6 inhibits the degradation of β-catenin, which regulates the expression of many genes. Receptor activation leads to the recruitment of the disheveled protein (Dvl). Dvl is then activated via phosphorylation, inducing the dissociation of GSK-3β from axin and causing GSK-3β to be deactivated. The deactivation of GSK-3β inhibits the degradation of β-catenin, allowing it to translocate into the nucleus. The nodes at which current therapies target the pathway are included as well. Created with BioRender.com.

Wnt binds to transmembrane receptor frizzled with the assistance of low-density lipoprotein receptor-related proteins 5 and 6 (LRP5/6), which are membrane protein [119]. The signal is then transduced to disheveled. Axin and disheveled are recruited to the cell membrane, leading to the inhibition of the glycogen synthase kinase (GSK)-3β. GSK-3β is responsible for regulating β-catenin proteasomal degradation [119]. When GSK-3β is inhibited, β-catenin accumulates in the cytoplasm and is then translocated to the nucleus to act as a transcription factor [119]. A diagram of the Wnt signaling pathway is shown in Figure 8.

#### 3.7.1. Wnt/β-Catenin Pathway and BC

Likely, Wnt signaling is constitutively activated via autocrine mechanisms [119]. Wnt mutations are less common in BC; however, 50% of BC cases show a high concentration of stabilized β-catenin and amplified disheveled expression. In 78% of malignant BC, Frizzled-related protein 1 (FRP1) is lost, causing poor prognosis. In vivo studies have demonstrated that activated β-catenin promotes TNBC and plays a role in HER-2 mammary tumors [119].

There are diverse categories of Wnt/ β– catenin targeted agents for cancer treatment. These treatment types include CBP inhibitors, disheveled inhibitors, Wnt antagonists, LRP5/6 inhibitors, CK1α agonists, and PORCN inhibitors [135]. PORCN inhibitor, GNF-6231, was shown to have potent inhibitor and antitumor activity in the BC mouse model [135]. It has also been reported that WNT974, a PORCN inhibitor, in combination with the PI3K inhibitor, buparlisib, inhibited BC stem cell proliferation [136]. In basal-like BC, an FDA-approved anti-helminthic agent, niclosamide, was shown to decrease levels of BC stem cells by reducing LRP6 and β- catenin concentrations [137]. Natural compounds such as gigantol have also been shown to inhibit WNT signaling in BC [138].

#### 3.7.2. Wnt/β-Catenin Pathway and Resistance

As previously stated, the induction of the EMT-like phenotype and the production of CSC are major mechanisms of drug resistance. It is well known that Wnt signaling regulates stemness in normal tissue [139]. In BC, Wnt1 has been shown to encourage the EMT-like phenotype through the upregulation of Twist [140]. It has also been demonstrated that overexpression of Twist and other EMT-related factors, such as snail, has increased CSC markers [141]. Other Wnt targets, such as ABCB1, CD44, CD24, and CD133, are also associated with CSC and resistance [142,143,144].

In mouse mammary glands and human BC cell lines, Wnt pathway activation has increased radiation resistance in progenitor cells. It is speculated that this is achieved by regulating stem and progenitor cell populations [119]. In human mammary epithelial cells, Wnt1 expression causes increased stem cell renewal and prevents apoptosis and senescence. β–catenin expression has been linked to chemoresistance in TNBC cells [145]. When knocked out in TNBC cells, β–catenin helped resensitize the cells to DOX and cisplatin [146]. Frizzled receptor overexpression can also induce DOX resistance [147]. Increased levels of nuclear and cytoplasmic β–catenin accumulation have also been shown to cause ABCB1 in BRCA-mutated TNBC cells, resulting in the efflux of PARPi [148]. An in vivo investigation found that after treatment with GDC-0941, a pan-PI3K inhibitor, the Wnt/beta-catenin pathway was activated, leading to resistance in TNBC cells [149]. Once treated with GDC-0941 and a Wnt inhibitor, LGK974, a resistance reversal was observed. Because of this, it is speculated that combination therapies that target the Wnt pathway may be beneficial in combating certain types of drug resistance.

### 3.8. HER-2

**Figure 9:**
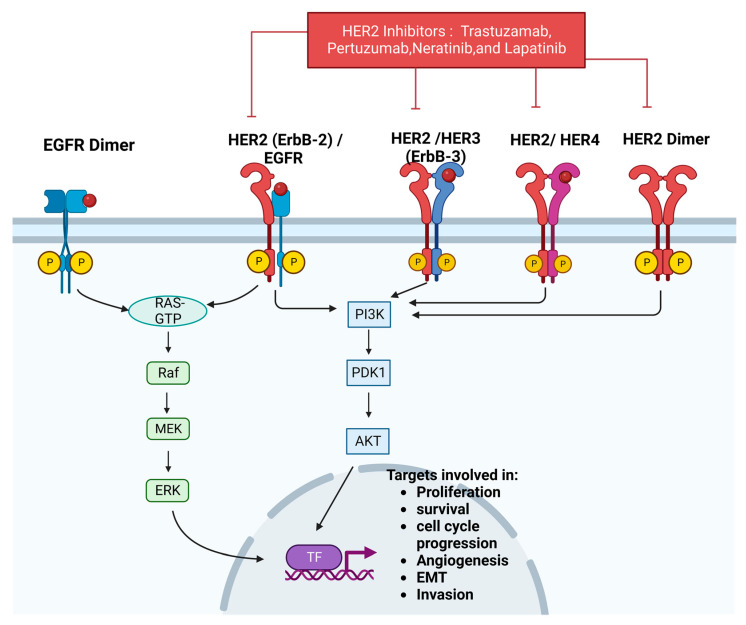
EGFR, HER2, HER3, HER4 signaling pathways. Created with Biorender.com.

Human epidermal growth factor (Her-2) is a tyrosine kinase receptor belonging to the ERBB family [150]. The ERBB family consists of four receptors, ErbB-2 (HER-2), epidermal growth factor receptor (EGFR), ErbB-3 (HER3), and ErbB-4 (HER4), that all consist of an extracellular ligand binding domain, a transmembrane domain, and an intracellular domain that includes a kinase domain [150]. The EGFR, HER2, HER3, and HER4 signaling pathways are shown in Figure 9. Because HER-2 cannot bind EGF-like ligands, Her-2 is activated via homodimerization and heterodimerization with other ERBB family receptors [151]. Once activated, tyrosine residues in the HER-2 intracellular domain become phosphorylated. This leads to the activation of downstream signaling pathways such as PI3K/AKT and Ras/MAPK, causing cell cycle proliferation, cell survival, invasion, and proliferation [119].

#### 3.8.1. HER-2 and BC

When BC cells have higher HER-2 expression than normal, they are referred to as HER-2 positive (HER-2+). HER-2+ accounts for about 20% of all BCs [151,152]. Her2 drives tumorigenesis by activating signaling pathways such as RAS/MAPK and PI3K/Akt [153]. Because of this, HER2 expression has been linked to cell proliferation, differentiation, angiogenesis, survival, invasion, and EMT [153]. An in vivo mouse model was used to show that E2F1-3 transcript expression is high in HER-2+ tumors [154]. E2F1 and E2F3 knockout mice exhibited protection against HER-2+ BC [154]. HER2+ BC is typically treated using monoclonal antibodies, kinase inhibitors, and antibody–drug conjugates [155]. Some standard HER2+ treatment options include trastuzumab, pertuzumab, neratinib, and lapatinib [26,155].

#### 3.8.2. HER-2 and Resistance

Although HER-2+ diagnosis has been linked to adverse health outcomes, targeted treatments have been produced using monoclonal antibodies and tyrosine kinase inhibitors [23]. Unfortunately, tumor progression is often seen after some time in these treatments because of resistance to the treatments [152]. HER-2 drug resistance has been shown to use mechanisms such as decreased drug binding, lack of HER-2 downregulation, dysregulation of apoptosis and the cell cycle, and immunodeficiency [152]. HER-2 expression prior to cancer production has also been linked to BC stem cell expansion [119]. In vivo and in vitro studies have demonstrated that anti-HER2 therapy resistance can occur due to CDK4/6-cyclin D1 expression [156]. When targeted with a CDK4/6 inhibitor, the BC was re-sensitized to ant-HER2 therapy [155,156]. Dysregulation of the PI3K/AKT/mTOR pathway has also been linked to trastuzumab resistance [155]. A phase III trial investigated the effects of using Everolimus, an mTOR inhibitor, in combination with trastuzumab and paclitaxel. The evidence showed that HER2- patients benefited more from adding Everolimus [157]. In a follow-up phase III trial, however, it was found that when patients previously treated with trastuzumab were later treated with Everolimus + trastuzumab, patient median progression-free survival (PFS) significantly increased [158].

### 3.9. EGFR

Epidermal growth factor receptor (EGFR), also known as ErbB1 or HER1, is one of four members of the epidermal growth factor receptor tyrosine kinase family [159]. The family also consists of ErbB2 (HER2), ErbB3 (HER3), and ErbB4 (HER 4). The receptors in this family are transmembrane single-chain glycoproteins that comprise a ligand-binding extracellular domain, a transmembrane domain, a tyrosine kinase domain, and a tyrosine-containing C-terminal tail [159]. EGFR can bind ligands and autophosphorylate the C-terminal tail through intracellular tyrosine domains [159]. The leucine-rich subdomains L1 and L2 interact with the ligand. Binding of the ligands epidermal growth factor (EGF) or transforming growth factor alpha (TGF-α) to the L1 and L2 domains leads to conformational changes that expose the dimerization loop, allowing for homo- and heterodimerization [159]. Receptor dimerization is crucial for the activation of the tyrosine kinase domains. Activation of these receptors can ultimately lead to the activation of several different pathways, such as the Ras/MAPK, PI3K/Akt, PLCγ1/PKC, STAT, and the Par6-atypical PKC pathways [159]. EGFR can lead to the protein kinase activation of c-Jun and MAPK activation via the activation of PLCγ, which ultimately leads to the regulation of cell proliferation [159]. STAT3, known to play an essential role in epithelial cell polarity and adhesion, binds to activated EGFR, which leads to the dimerization and nuclear translocation of STAT3.

#### 3.9.1. EGFR and BC

EGFR overexpression is linked to BC recurrence, metastasis, and decreased survival [160]. A study found that the metastatic phenotypes of tumor cells are promoted by constitutively active forms of EGFR [161]. Another study found that EGFR overexpression resulted in higher rates of lung metastasis in mammary tumors when compared to tumors with lower EGFR expression [162]. A study examining the underlying mechanism behind EGFR expression and metastasis in BC cells showed that hormonally upregulated neu-associated kinase (HUNK) directly phosphorylates EGFR at T654, promoting metastasis [160].

#### 3.9.2. EGFR and Resistance

Although EGFR has been linked with poor clinical outcomes; EGFR-related inhibitors have not shown significant efficacy. BCs overexpressing EGFR and having increased metastasis rates are resistant to EGFR inhibitors [163]. One known mechanism for the resistance to EGFR-directed therapies is the activation of downstream or parallel pathways. This allows for tumor growth, although there is a lack of EGFR signaling. Mutant EGFR tumors can activate the PI3K/AKT pathway, promoting cell survival via the activation of ErbB3 [159].

BC cells resistant to the EGFR tyrosine kinase inhibitor, gefitinib, exhibited persistent MAPK signaling, suggesting that this pathway is also related to anti-EGFR treatment resistance [164]. Combining therapies targeting multiple pathways has been recommended to be more effective in overcoming tyrosine kinase inhibitor-related resistance.

Using in vitro and in vivo experiments, it has been demonstrated that rationally designed drug combinations are efficient and selective in inhibiting tumor regrowth. They can prevent the creation of evolutionary pressure, resulting in drug resistance [165].

## 4. Conclusions and Future Paradigm of BC Resistance and Treatment

BC is the leading cause of death in women internationally. BC heterogeneity and the complexity of resistance make it unlikely that one type of therapy can cure all types of cancer. With further research, combination therapies aimed at the previously mentioned drug targets can potentially treat BC more effectively and bypass resistance.

## Figures and Tables

**Table 1:** Past and current BC clinical trials related to HER2, PARP, EGFR, aryl hydrocarbon receptor (AhR), inducible nitric oxide (iNOS), and Wnt.

Intervention/Therapy	Target Cancer Subtype	Clinical Trial Phase	Type	Status	Trial ID Reference (Clinicaltrials.gov)
KU 0059436 (olaparib), a PARP inhibitor	BRCA1- or BRCA2-positive advanced BC	Phase II	Treatment	Active	NCT00494234
Preoperative combination of letrozole, everolimus, and TRC105	Postmenopausal hormone-receptor positive and Her2 BC	Phase I	Treatment	Active	NCT02520063
CDK4/6-inhibitor or chemotherapy, in combination with endocrine therapy	Advanced BC	Phase II	Treatment	Recruiting	NCT03227328
LGK974 in patients with malignancies dependent on Wnt ligands	TNBC	Phase I	Treatment	Recruiting	NCT01351103
Anti-EGFR-immunoliposomes loaded with DOX	Advanced triple negative EGFR positive BC	Phase II	Treatment	Active	NCT02833766
Comparing alpelisib and fulvestrant versus chemotherapy as maintenance therapy	PIK3CA mutated advanced BC	Phase II	Treatment	Active	NCT03386162
Peritumoral adipose tissue sample analyzing the concentrations of 46 persistent organics pollutants	Breast tumor patients (benign, malignant with and without lymph node metastasis)	N/A	Other	Completed	NCT03788187
Seviteronel in combination with chemotherapy	Androgen-receptor-positive metastatic TNBC	Phase I, Phase II	Treatments	Not yet recruiting	NCT04947189
